# Earlier anti-TNF therapy reduces the risk of malnutrition associated with alterations in body composition in patients with Crohn’s disease

**DOI:** 10.3389/fnut.2023.1114758

**Published:** 2023-02-07

**Authors:** Yuexin Wang, Danhua Yao, Yining He, Qi He, Yousheng Li

**Affiliations:** ^1^Department of General Surgery, Shanghai Jiao Tong University School of Medicine Affiliated Ninth People's Hospital, Shanghai, China; ^2^Biostatistics Office of Clinical Research Unit, Shanghai Jiao Tong University School of Medicine Affiliated Ninth People's Hospital, Shanghai, China

**Keywords:** Crohn’s disease, body composition analysis, bioelectrical impedance analysis, anti-TNF therapy, malnutrition

## Abstract

**Background:**

Anti-TNF therapy has been found to exert an influence on long-term nutritional status and even reverse malnutrition in patients with Crohn’s disease.

**Aims:**

to observe the effect of anti-TNF therapy on nutritional status in patients with Crohn’s disease, investigate the correlation between the timing of anti-TNF therapy and the human body composition and examine independent body composition factors for predicting malnutrition in these patients.

**Methods:**

This was a retrospective study of 115 patients with Crohn’s disease. Body composition parameters were assessed by bioelectrical impedance analysis. The nutritional status of the patients was determined by NRS2002 and MNA.

**Results:**

The BMI, BFMI, FFMI, BCMI, SMI, BMC, intracellular water, protein and BMR were significantly lower in patients without any biologic agents (*p* < 0.05). Negative correlations were found between BMC, intracellular water, extracellular water, protein and BMR and the interval between the first symptom and first dose by Spearman’s correlation analysis (*r* < 0, *p* < 0.05). Low BMI (OR 0.602, 95% CI 0.434–0.836, *p* = 0.002), low FFMI (OR 0.678, 95% CI 0.507–0.906, *p* = 0.009), and low BCMI (OR 0.564, 95% CI 0.367–0.868, *p* = 0.009) were independent risk factors for malnutrition in Crohn’s disease patients. Anti-TNF therapy tended to reduce the malnutrition probability as assessed by Cox regression analysis (OR: 0.217, 95% CI 0.057–0.821, *p* = 0.024).

**Conclusion:**

Body composition analysis is predictive of malnutrition in patients with Crohn’s disease. Early application of anti-TNF therapy significantly affected skeletal muscle mass, fat mass and bone mineral content, supporting their long-term nutritional status and reducing their probability of malnutrition.

## 1. Introduction

Crohn’s disease (CD), as a progressive and relapsing inflammatory bowel disease, has a high frequency of associated nutritional and metabolic issues (1–3). The prevalence of malnutrition is up to 85% in patients with CD (4) Lesions of CD are scattered throughout the whole gastrointestinal tract, mainly in the small intestine, contributing to the malabsorption of various nutrients (4). Due to decreased oral food intake, energy metabolism disorders and chronic nutritional depletion, the body composition of these patients, including body fat, skeletal muscle and bone mineral, was always on a declining trend (5) and was associated with a range of secondary manifestations, such as sarcopenia (6, 7) and osteopenia (8, 9). Therefore, nutrition assessment and support have long been one of the primary treatments for CD patients.

At present, in addition to some universal nutrition assessment scales, bioelectrical impedance analysis (BIA) is being more widely used to assess the nutritional status of patients. It can precisely measure the human body composition, which assists in the development of reasonable therapeutic options targeting the consumption of patients suffering from malnutrition in an efficient, painless, and noninvasive way (10, 11). In CD patients, multiple body composition analysis was found to perform better for the screening and assessment of malnutrition than nutrition assessment scales or laboratory data. Moreover, alterations in body composition were significantly associated with adverse outcomes of severe CD, whereas usual nutritional assessment was not (12).

Currently, the use of biologic agents has provided significant advances in the remission of Crohn’s disease (13, 14). Despite a 10–30% primary nonresponse and 5% secondary loss of response per patient-year (15), biologic agents targeting TNF-α, including infliximab and adalimumab, make up the largest fraction of biologic therapies currently applied in clinical practice to CD. Previous studies have demonstrated that earlier implementation of anti-TNF therapy is associated with a reduced rate of surgery (16) and slower progression of bowel damage (17), affecting long-term outcomes (18–20).

In CD patients, the severity of malnutrition is largely dependent on the activity, duration and extent of bowel inflammation, which is regulated by proinflammatory cytokines, particularly TNF-α (5). Enteral nutrition therapy is commonly considered the primary treatment to maintain the nutritional status of individuals (21). However, few studies have reported the benefit of early anti-TNF therapy in relieving malnutrition.

This study mainly aimed to observe the effect of anti-TNF therapy on the nutritional status of CD patients, to investigate the correlation between the timing of anti-TNF therapy and the human body composition and to examine independent body composition factors for predicting malnutrition.

## 2. Materials and methods

### 2.1. Patient population

Patients diagnosed with Crohn’s disease at the Department of General Surgery, Shanghai Jiao Tong University School of Medicine Affiliated Ninth People’s Hospital between December 2020 and February 2022 were selected from the institutional database. All enrolled subjects fulfilled the following criteria: (a) ≥18 years old, (b) with a confirmed diagnosis of Crohn’s disease based on clinical, radiological, and histological investigation, (c) never received biologic treatment or received consecutive anti-TNF therapy ≥6 months, and (d) underwent multifrequency bioimpedance analysis. Patients were excluded from this study if (a) the interval between BIA and the last dose >3 months in the subgroup of anti-TNF therapy, (b) with only perianal diseases or extraintestinal manifestations, or (c) had a history of failure of the vital organs or other severe illness that might impact the weight and body composition.

### 2.2. Data collection

The following patient characteristics were collected from the medical records: demographic data including sex, age, height, weight, body mass index, disease duration, location of the disease and disease behaviour according to the Montreal classification; nutritional risk according to the nutrition risk screening 2002 (NRS2002); and the presence of malnutrition according to the mini nutritional assessment (MNA). Laboratory data, including haemoglobin concentration (Hb), haematocrit (Hct), white blood cell counts (WBC), serum levels of C-reactive protein (CRP), albumin (ALB), and erythrocyte sedimentation rate (ESR), were recorded. Furthermore, data on medications that the patients received, such as the type of anti-TNF therapy, including infliximab and adalimumab, the duration of administration, and the interval between the first onset of symptoms and the first dose of biologics, were recorded. During the study period, the type of concomitant therapy received by all patients, including 5-aminosalicylic acid (5-ASA), salazosulfapyridine (SASP), steroids, and immunomodulators, was registered.

### 2.3. Body composition assessment

The human body composition parameters were measured by bioelectrical impedance analysis (BIA) using the InBody S10 analyser (Biospace Co., Ltd., Seoul, Korea), which evaluated thirty electrical impedance measurements in 6 different frequencies (1 kHz, 5 kHz, 50 kHz, 250 kHz, 500 kHz, and 1,000 kHz) and fifteen reactance and phase angle measurements in 3 different frequencies (5 kHz, 50 kHz, and 250 kHz) performed on five segmental sections of the human body by an 8-point contact electrode method. The patients received the BIA measurements during standardized procedures after lying prone for 10 min to redistribute their body water after an overnight fast and with an empty bladder at an ambient temperature of 20–25°C.

Each BIA parameter was calculated based on a fixed relationship between the body composition mass and height. Fat-free mass index (FFMI) = fat-free mass/height2, body fat mass index (BFMI) = (body weight-fat-free mass)/height2, body cell mass index (BCMI) = body cell mass/height2, skeletal muscle mass index (SMI) = appendicular skeletal muscle mass/height2. In addition, bone mineral content (BMC), protein, intracellular water, extracellular water and basal metabolic rate (BMR) were available directly from an InBody S10 analyser.

### 2.4. Statistical analysis

SPSS version 26 (IBM Software Group, San Francisco, California, United States) was used for the statistical analysis. Numerical data consistent with a normal distribution are expressed as the means ±standard deviations (SDs), and those not normally distributed are presented as medians with interquartile ranges (IQRs). Categorical data were expressed as frequencies with percentages. The t-test or chi-squared test was utilized for comparisons of general characteristics and body composition parameters. Spearman’s correlation analysis was used to explore a possible correlation between the timing of anti-TNF therapy and the body composition. The risk factors for malnutrition were evaluated by univariate logistic regression analysis. A Cox proportional hazards model was established to predict the long-term malnutrition probability for patients with or without anti-TNF therapy.

## 3. Results

### 3.1. General characteristics

**Figure 1:**
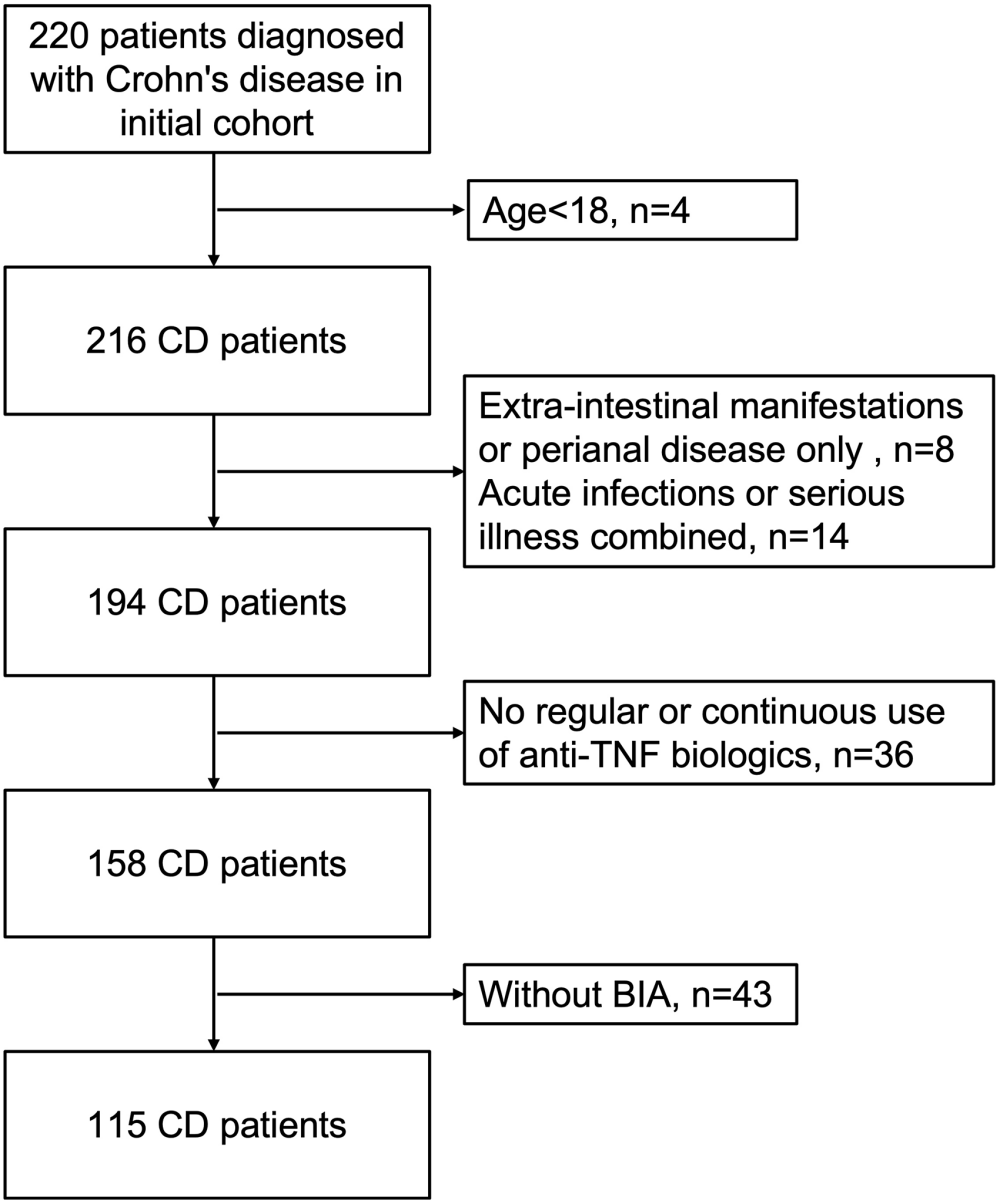
Study flow chart explaining the CD patients’ selection.

A total of 220 patients diagnosed with Crohn’s disease between December 2020 and February 2022 at our centre. Of these, four patients were under 18 years of age, 43 patients were without bioelectrical impedance analysis, 36 patients did not receive regular or continuous use of anti-TNF biologics, eight patients were with extra-intestinal manifestations or perianal disease only, and 14 patients were with acute infections, trauma or other serious illnesses in combination (Figure 1). Therefore, a total of 115 patients with Crohn’s disease were included in this study (77 men, median age: 37.30 ± 10.80 years), with a mean disease duration of 84.48 ± 63.10 months. Among these subjects, 71 had received consecutive anti-TNF therapy for over 6 months (48 men, mean age: 35.76 ± 10.11 years), and 44 patients had never received any biological treatment (29 men, mean age: 39.77 ± 11.52 years). In addition, there were 70 patients treated with infliximab, and 4 were treated with adalimumab (3 of these 4 patients were given infliximab and adalimumab in succession) in the anti-TNF therapy group. Overall, the baseline weight and BMI of patients with anti-TNF therapy were significantly higher (weight, *p* = 0.002, BMI, *p* = 0.001). Low BMI (<18.5 kg/m2) occurred in 35 (30.43%) patients, 14 (19.72%) in the anti-TNF therapy group and 21 (47.73%) in the other group. Other than weight and BMI, there were no significant differences between the two groups of patients for any baseline characteristics. The patient characteristics are listed in Table 1.

**Table 1:** Baseline characteristics of 115 CD patients enrolled.

Characteristics	All	With anti-TNF therapy	Without anti-TNF therapy	*p*-Value
	(*n* = 115)	(*n* = 71)	(*n* = 44)	
Sex				0.851
Male	77 (66.96)	48 (67.61)	29 (65.91)	
Female	38 (33.04)	23 (32.39)	15 (34.09)	
Age at the time of BIA analysis (year)	37.30 ± 10.80	35.76 ± 10.11	39.77 ± 11.52	0.053
Disease duration (m)	84.48 ± 63.10	82.31 ± 53.84	87.98 ± 76.27	0.668
Height (m)	167.69 ± 8.57	168.35 ± 8.77	166.61 ± 8.23	0.292
Weight (kg)	56.92 ± 11.16	59.40 ± 11.14	52.92 ± 10.08	0.002*
BMI (kg/m^2^)	20.17 ± 3.02	20.90 ± 2.98	18.99 ± 2.74	0.001*
CDAI	187.28	188.10	185.91	0.914
**Montreal classification**				
A1	1 (0.87)	1 (1.41)	0 (0.00)	0.263
A2	86 (74.78)	56 (78.87)	30 (68.18)	
A3	28 (24.35)	14 (19.72)	14 (31.82)	
L1	36 (31.30)	20 (28.17)	16 (36.36)	0.267
L2	7 (6.09)	2 (2.82)	5 (11.36)	
L3	72 (62.61)	49 (69.01)	23 (52.27)	
L4	10 (8.70)	6 (8.45)	4 (9.09)	
B1	21 (18.26)	13 (18.31)	8 (18.18)	0.668
B2	79 (68.70)	49 (69.01)	30 (68.18)	
B3	40 (34.78)	27 (38.03)	13 (29.55)	
P	48 (41.74)	29 (40.85)	19 (43.18)	0.805
**Laboratory data**				
Hb (g/L)	112.92 ± 21.15	110.47 ± 20.17	116.40 ± 22.26	0.176
HCT	0.34 ± 0.06	0.34 ± 0.05	0.36 ± 0.06	0.151
WBC (*10^9^/L)	7.33 ± 4.80	7.14 ± 5.25	7.61 ± 4.14	0.641
CRP (mg/L)	33.31 ± 47.23	35.49 ± 49.07	30.01 ± 44.79	0.597
ALB (g/L)	37.71 ± 9.87	37.25 ± 9.86	38.38 ± 9.99	0.597
ESR (mm/h)	21.29 ± 18.19	19.89 ± 17.95	22.91 ± 18.63	0.497
Biologic agents				
Infliximab	70 (60.87)	70 (98.59)	–	
Adalimumab	4 (3.48)	4 (5.63)	–	
Duration of administration (m)	10 [0–25]	19 [10–31]	–	
Interval between first symptom and first dose (m)	34 [12–108]	34 [12–108]	–	
**Concomitant therapy**				
5-ASA	69 (60.00)	42 (59.15)	27 (61.36)	0.814
SASP	9 (7.83)	5 (7.04)	4 (9.09)	0.691
Steroids	27 (23.48)	18 (25.35)	9 (20.45)	0.547
Immunomodulators	60 (52.17)	40 (56.34)	20 (45.45)	0.256

### 3.2. Comparison of body composition parameters between patients with and without anti-TNF therapy

The body composition parameters measured by bioelectrical impedance analysis are described in Table 2. In general, the comparison demonstrated that the median parameters of all body compositions in the anti-TNF therapy group were higher than those in the other group, although the differences for extracellular water and BMR were not statistically significant. Specifically, the BMI, FFMI, BFMI, SMI, BCMI, BMC, intracellular water and protein of the two groups showed remarkable differences (*p* < 0.05). Of these, the SMI showed the most significant difference between the two groups (7.67 ± 1.31 versus 7.04 ± 1.14, *p* = 0.010), except for BMI. Regarding fat mass, the BFMI and FFMI of patients with anti-TNF therapy were both significantly higher than those without. However, the BFMI of patients who did not receive anti-TNF therapy showed a more significant decline (BFMI, 4.09 ± 2.17 versus 3.08 ± 2.06, *p* = 0.015; FFMI, 16.81 ± 2.46 versus 15.88 ± 1.90, *p* = 0.033). These results indicated that anti-TNF therapy might influence both the fat and skeletal muscle mass.

**Table 2:** Comparison of body composition measured by bioelectrical impedance analysis.

Variables	With anti-TNF therapy	Without anti-TNF therapy	*p*-value
	*n* = 71	*n* = 44	
BMI (kg/m^2^)	20.90 ± 2.98	18.99 ± 2.74	0.001*
FFMI (kg/m^2^)	16.81 ± 2.46	15.88 ± 1.90	0.033*
BCMI (kg/m^2^)	11.02 ± 1.67	10.33 ± 1.35	0.024*
BFMI (kg/m^2^)	4.09 ± 2.17	3.08 ± 2.06	0.015*
SMI (kg/m^2^)	7.67 ± 1.31	7.04 ± 1.14	0.010*
BMC (kg)	2.81 ± 0.69	2.57 ± 0.51	0.049*
Intracellular water (L)	22.02 ± 4.81	20.22 ± 4.17	0.043*
Extracellular water (L)	13.16 ± 2.63	12.40 ± 2.28	0.117
Protein (kg)	9.53 ± 2.07	8.76 ± 1.79	0.044*
BMR (kcal)	1408.83 ± 221.27	1355.89 ± 237.37	0.228

BMI, body mass index; FFMI, fat-free mass index; BFMI, body fat mass index; BCMI, body cell mass index; SMI, skeletal muscle mass index; BMC, bone mineral content; BMR, basal metabolic rate.

### 3.3. Correlations between the body composition parameters and the timing of anti-TNF therapy

**Figure 2:**
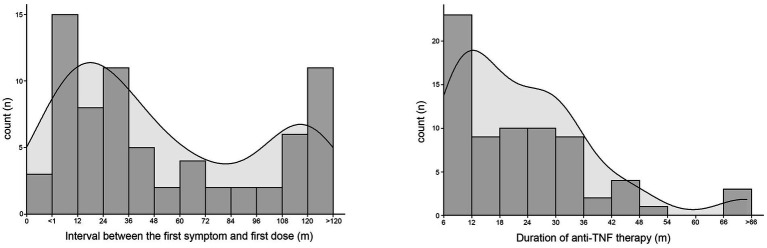
The number of patients who received anti-TNF therapy were divided by an interval of one year according to the interval between the first onset of symptoms and the first dose of biologics or the dosing time of biologics. The corresponding density curve are illustrated in each figure.

According to the timing and duration of anti-TNF therapy, 71 patients who were treated with infliximab or adalimumab were divided by an interval of 1 year. The number of patients in each subgroup and the corresponding density curve are illustrated in Figure 2. Among these patients, the median interval between the first symptom and the first dose was 34 [12–108] months, and the median duration of administration was 19 [10–31] months. The timing of anti-TNF therapy was negatively correlated with BMC (*r* = −0.249, *p* = 0.036), intracellular water (*r* = −0.245, *p* = 0.039), extracellular water (*r* = −0.260, *p* = 0.028), protein (*r* = −0.245, *p* = 0.039) and BMR (*r* = −0.248, *p* = 0.037), which suggested that when there was a longer duration between the first symptoms to the initial administration of anti-TNF agents, these body composition parameters would show a lower value (Table 3).

**Table 3:** Correlations between body composition parameters and timing of anti-TNF therapy.

	Interval between first symptoms and first dose		Duration of administration	
	*r*	*p-*Value	*r*	*p-*Value
BMI	−0.159	0.185	0.025	0.833
FFMI	−0.170	0.157	0.121	0.314
BCMI	−0.172	0.152	0.122	0.309
BFMI	0.019	0.877	−0.094	0.436
SMI	−0.190	0.113	0.136	0.257
BMC	−0.249	0.036*	0.069	0.568
Intracellular water	−0.245	0.039*	0.118	0.329
Extracellular water	−0.260	0.028*	0.138	0.250
Protein	−0.245	0.039*	0.118	0.328
BMR	−0.248	0.037*	0.122	0.312

BMI, body mass index; FFMI, fat-free mass index; BFMI, body fat mass index; BCMI, body cell mass index; SMI, skeletal muscle mass index; BMC, bone mineral content; BMR, basal metabolic rate.

In this study, no significant correlations were found between the dosing time of anti-TNF and the human body composition parameters (*p* > 0.05) (Table 3).

### 3.4. Body composition factors for high nutritional risk in patients with anti-TNF therapy

**Figure 3:**
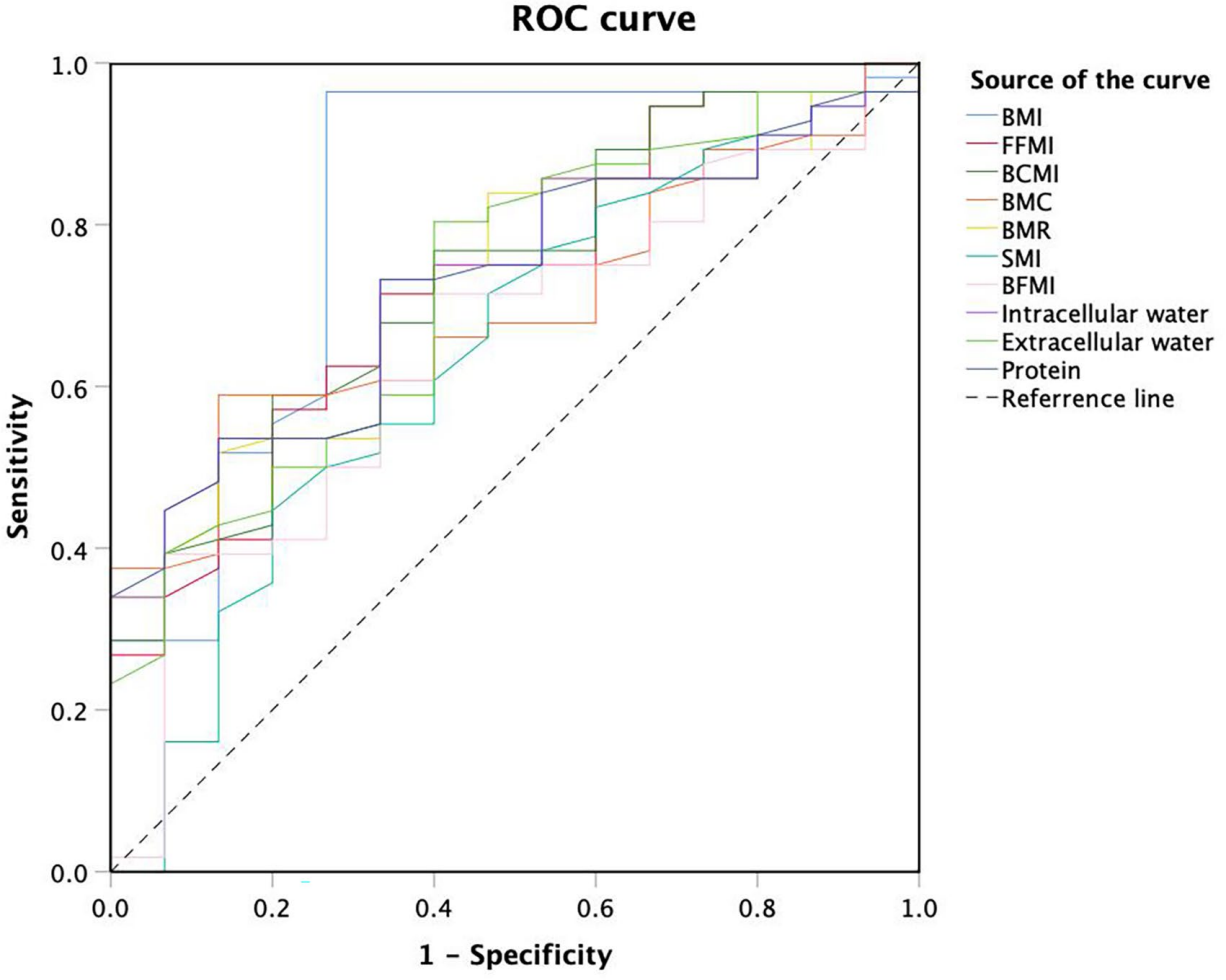
Receiver operating characteristic curve analysis of body composition cut-off values for malnutrition.

In 71 patients with anti-TNF therapy, 15 (21.13%) scored ≥3 as assessed by NRS2002, suggesting a high risk of malnutrition and the need for nutritional support. Univariate analysis of body composition factors for nutritional risk was performed in 71 patients treated with anti-TNF therapy (Table 4). Except for SMI and BFMI, all body composition parameters were found to notably increase the risk of malnutrition. BMI (OR 0.602, 95% CI 0.434–0.836, *p* = 0.002), FFMI (OR 0.678, 95% CI 0.507–0.906, *p* = 0.009), and BCMI (OR 0.564, 95% CI 0.367–0.868, *p* = 0.009) were the most independent risk factors, with *p* values under 0.01. ROC analysis was performed to determine the cut-off values for body composition (Table 5; Figure 3). Among all of the parameters, the cut-off values of BMI and BMR had both high sensitivity and specificity: BMI, cut-off value 18.45 kg/m2, sensitivity 96.4%, specificity 73.3%, Youden index 0.697, AUC 0.819; BMR, cut-off value 1301.5 kcal, sensitivity 73.2%, specificity 66.7%, AUC 0.728.

**Table 4:** Univariate analysis of body composition associated with nutritional risk.

	OR	95%CI	*p*-Value
BMI	0.602	0.434–0.836	0.002*
FFMI	0.678	0.507–0.906	0.009*
BCMI	0.564	0.367–0.868	0.009*
BFMI	0.807	0.582–1.120	0.200
SMI	0.760	0.477–1.212	0.249
BMC	0.257	0.085–0.782	0.017*
Intracellular water	0.832	0.718–0.964	0.014*
Extracellular water	0.721	0.555–0.937	0.014*
Protein	0.651	0.463–0.915	0.013*
BMR	0.996	0.993–0.999	0.013*

BMI, body mass index; FFMI, fat-free mass index; BFMI, body fat mass index; BCMI, body cell mass index; SMI, skeletal muscle mass index; BMC, bone mineral content; BMR, basal metabolic rate.

**Table 5:** Cut-off values of body composition for nutritional risk.

	Cut-off	Sensitivity	Specificity	Youden index
BMI (kg/m^2^)	18.45	0.964	0.733	0.697
FFMI (kg/m^2^)	15.86	0.714	0.667	0.381
BCMI (kg/m^2^)	10.915	0.589	0.800	0.389
BFMI (kg/m^2^)	4.645	0.393	0.933	0.326
SMI (kg/m^2^)	7.05	0.714	0.533	0.247
BMC (kg)	2.74	0.589	0.867	0.456
Intracellular water (L)	22.9	0.536	0.867	0.403
Extracellular water (L)	11.2	0.804	0.600	0.404
Protein (kg)	9.9	0.536	0.867	0.403
BMR (kcal)	1301.5	0.732	0.667	0.667

BMI, body mass index; FFMI, fat-free mass index; BFMI, body fat mass index; BCMI, body cell mass index; SMI, skeletal muscle mass index; BMC, bone mineral content; BMR, basal metabolic rate.

### 3.5. Probability of malnutrition associated with anti-TNF therapy according to body composition

**Figure 4:**
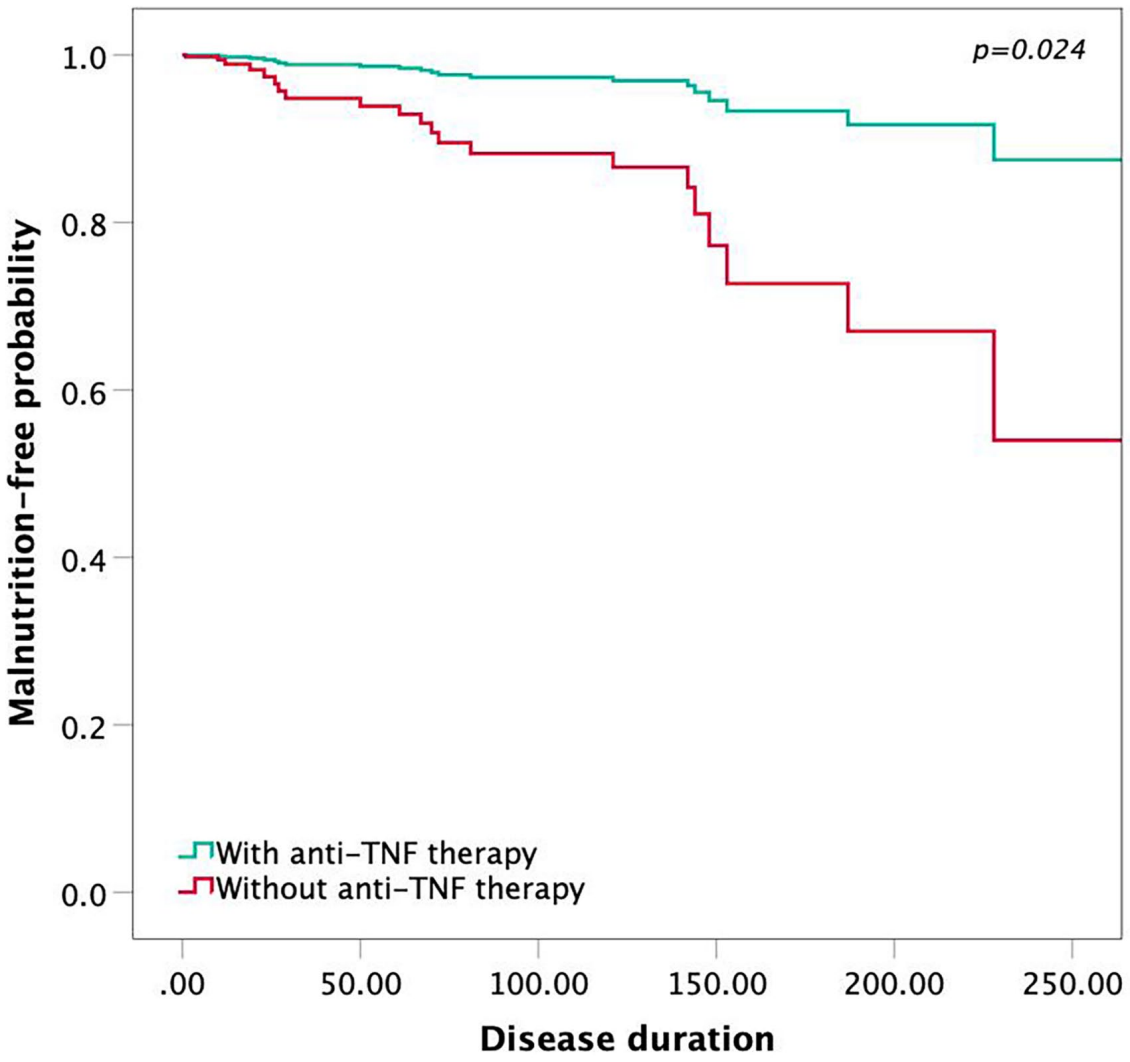
The Cox proportional hazards model showing the malnutrition-free probability by anti-TNF therapy.

Twenty-two of 115 (19.13%) patients had malnutrition, and fifty-three (46.09%) were at risk of malnutrition, as evaluated by the MNA. Among all malnutrition patients, 4 (5.63%) patients received anti-TNF therapy, while 18 (40.91%) did not. In a multivariate analysis by the Cox regression model, patients with anti-TNF therapy showed a higher malnutrition-free probability according to the MNA (Figure 4; *p* = 0.024). Low BMI, BMC and BFMI were identified as risk factors for the presence of malnutrition (BMI, *p* = 0.019; BCMI, *p* = 0.035; BMC, *p* = 0.014; BFMI, *p* = 0.007; extracellular water, *p* = 0.026), suggesting that they would predict the long-term nutritional status of CD patients treated with anti-TNF therapy.

## 4. Discussion

To our knowledge, this is the first study to investigate the correlation between the timing of anti-TNF therapy and body composition analysis in patients with CD. Currently, bioelectrical impedance analysis has become a widespread method of measuring the human body composition to reflect the nutritional status of patients in clinical practice (22). Our correlation analysis revealed that the timing of anti-TNF therapy influenced the patient’s body composition. The interval between the first onset of CD symptoms and the first dose of anti-TNF therapy was negatively correlated with BMC, intracellular water, extracellular water, protein and BMR, suggesting that earlier anti-TNF therapy may improve these parameters in CD patients. Meanwhile, this study found that low BMI, BCMI, FFMI, BMC, BMR, protein, intracellular water and extracellular water were prognostic markers of malnutrition by univariate analysis. Consequently, we supposed that earlier anti-TNF therapy was effective in reducing the potential for malnutrition. Additionally, in accordance with our Cox proportional hazards model, patients without anti-TNF therapy tended to have a higher probability of malnutrition.

Weight loss and reduction in body composition can occur in both ulcerative colitis and Crohn’s disease, but it has been reported to have a higher presence in CD patients (23). Consistent with the systematic review of Bryant et al. (23), approximately one-third of patients suffered a low BMI in our cohort. Moreover, the rate of low BMI in patients who did not receive anti-TNF therapy was approximately 2.5 times higher than that in those who received anti-TNF therapy (40.73% versus 19.72%). A recent study discovered that insulin-like growth factor-1 (IGF-1) in the serum of adult CD patients was downregulated by adalimumab and that IGF-1 treatment contributed to the restoration of the mucosal barrier, attenuating weight loss and shortening of the colon (24), which may explain the retention of lean mass by anti-TNF therapy.

In our study, all body composition parameters were higher in patients with anti-TNF therapy, although the increases in extracellular water and BMR were not statistically significant. Regarding fat mass, we found significant differences in BFMI and FFMI between the two groups. Fat wrapping or “creeping fat” has long been identified as a marker predicting the activity of CD (25), which is positively associated with muscle hypertrophy, fibrosis, transmural inflammation and intestinal stricture (26–28). Despite the reduction in total fat mass, the ratio of visceral adipose tissue to total abdominal adipose tissue is much higher due to the conversion of subcutaneous fat into visceral fat (25). Furthermore, a previous study by Shen et al. demonstrated correlations between the low visceral fat area in Asian patients and mucosal healing, as well as positive outcomes after anti-TNF therapy (29). Hence, further studies are required to measure visceral fat and subcutaneous fat to investigate the effect of anti-TNF therapy on fat wrapping. Likewise, regarding skeletal muscle and bone mineral content, we also discovered a lower SMI and BMC in patients without anti-TNF therapy. To date, many published studies have examined the effects of anti-TNF therapy on skeletal muscle and bone loss measured by dual-emission X-ray absorptiometry or CT scans (27, 30, 31). Sarcopenia and osteopenia, defined as a skeletal muscle index or bone mineral density lower than the cut-off values, were evident in 12 and 30% of CD patients, respectively (32, 33), and are known to be predictive markers of adverse outcomes (12, 27, 34, 35). In a clinical trial by Subramaniam et al., infliximab was found to reverse inflammatory sarcopenia, consistent with our results (36).

Early anti-TNF therapy is known to play a pivotal role in improving the long-term outcomes of Crohn’s disease. Oh et al. demonstrated that late anti-TNF or immunomodulator therapy tended to correlate with a higher risk of intestinal surgery, behavioural progression and stricturing complications in Asian patients (19). In addition, Panchal et al. showed that earlier introduction of biologics was associated with a slower rate of progression (17). Our study also demonstrated that there was a strong correlation between the timing of anti-TNF therapy and body composition components, especially bone mineral content, suggesting that delaying anti-TNF therapy may increase the possibility of osteopenia. However, no significant correlation was found between the duration of anti-TNF treatment and body composition components. Here, we present a possible role of the disease activity to explain this result. All patients in the anti-TNF therapy group received consecutive biologic treatment for more than 6 months. In other words, most of these CD patients were in clinical remission and without large fluctuations in body weight. Considering that the severity of malnutrition largely depends on disease activity, it is reasonable to believe that patients in remission are expected to be in a stable nutritional status without remarkable alterations of body composition components.

There are several limitations of our study. First, the small sample size and subjects selected from a single centre may account for some of the insignificant results. As a surgery department, a vast majority of our inpatients suffered more severe manifestations of CD, such as fistula and abscess, and exhibited more serious phenotypes. Compared to the total population of Crohn’s disease patients, our patients may have a worse nutritional status and lower body composition parameters in general. Second, our retrospective study, as opposed to prospective cohort studies, did not provide a cause-and-effect relationship between body composition and anti-TNF therapy. These findings merely articulated a significant difference and association, which offered potential insight into therapeutic strategies for CD. However, it is a strength that the impacts of changes in the disease behaviour and location according to the Montreal classification caused by CD progression during the study period would be avoided. Third, indicators to evaluate the efficiency of anti-TNFα agents, such as serum infliximab trough concentrations and anti-infliximab antibody, were not taken into consideration when investigating the correlation between treatment and body composition. The majority of patients were under maintenance therapy, but there may be a few individuals who respond less well to anti-TNFα agents. Further research could be carried out to include these indicators in the analysis. Finally, although there were some patients with missing laboratory data, this is not supposed to exert much of influences on our study.

In conclusion, body composition analysis is predictive of malnutrition in patients with Crohn’s disease. Early application of anti-TNF therapy significantly affected the skeletal muscle mass, fat mass and bone mineral content, supporting their long-term nutritional status and reducing the probability of malnutrition in these patients.

## Data availability statement

The raw data supporting the conclusions of this article will be made available by the authors, without undue reservation.

## Ethics statement

Ethical review and approval was not required for the study on human participants in accordance with the local legislation and institutional requirements. The patients/participants provided their written informed consent to participate in this study.

## Author contributions

YW, QH, and YL contributed to conception and design of the study. DY organized the database. YH performed the statistical analysis. YW wrote the first draft of the manuscript. YW, DY, QH, and YL wrote sections of the manuscript. All authors contributed to the article and approved the submitted version.

## Conflict of interest

The authors declare that the research was conducted in the absence of any commercial or financial relationships that could be construed as a potential conflict of interest.

## Publisher’s note

All claims expressed in this article are solely those of the authors and do not necessarily represent those of their affiliated organizations, or those of the publisher, the editors and the reviewers. Any product that may be evaluated in this article, or claim that may be made by its manufacturer, is not guaranteed or endorsed by the publisher.
